# Circulating CD14^+^ HLA‐DR ^‐/low^ myeloid‐derived suppressor cells in leukemia patients with allogeneic hematopoietic stem cell transplantation: novel clinical potential strategies for the prevention and cellular therapy of graft‐versus‐host disease

**DOI:** 10.1002/cam4.688

**Published:** 2016-04-25

**Authors:** Jin Yin, Chunyan Wang, Min Huang, Xia Mao, Jianfeng Zhou, Yicheng Zhang

**Affiliations:** ^1^ Department of HematologyTongji HospitalTongji Medical CollegeHuazhong University of Science and TechnologyWuhan430030China; ^2^ Clinical LaboratoryTongji HospitalTongji Medical CollegeHuazhong University of Science and TechnologyWuhan430030China

**Keywords:** Allogenetic hematopoietic stem cell transplantation, graft‐versus‐host disease, myeloid‐derived suppressor cells

## Abstract

Myeloid‐derived suppressor cells (MDSCs) are a heterogeneous cell population that includes immature myeloid cells and the progenitor cells of macrophages, dendritic cells (DCs), monocytes, and neutrophils. The expansion and functional importance of MDSCs in patients with cancer and noncancer pathogenic conditions has been recognized. As a result, there has been growing interest in understanding their roles in acute graft‐versus‐host disease (aGVHD) after allogenetic hematopoietic stem cell transplantation (allo‐HSCT). In order to evaluate possible effects of MDSCs on aGVHD development and clinical outcomes, this study systematically detected the dynamic changes of MDSCs accumulation in patients during the first 100 days after allo‐HSCT, and investigated the levels of other cell types and relative cytokines during MDSCs accumulation. Results showed that accumulation of MDSCs in the graft and in peripheral blood when engraftment might contribute to patients' overall immune suppression and result in the successful control of severe aGVHD and long‐term survival without influence on risk of recurrence after allo‐HSCT. But MDSCs levels in the graft had more favorable predictive abilities. Furthermore, MDSCs proportion significantly increased in patients developing aGVHD after allo‐HSCT. It might be caused by secondary inflammatory response, especially related to high concentrations of IL‐6 and TNF‐*α*. But this accumulation would not be able to counterbalance the aggravation of aGVHD and would not have influence on clinical outcomes and risk of relapse. Overall, MDSCs might be considered as potential new therapeutic option for aGVHD and achieve long‐term immunological tolerance and survival.

## Introduction

Myeloid‐derived suppressor cells (MDSCs), originally described in cancer patients, are a heterogeneous cell population that includes immature myeloid cells and the progenitor cells of macrophages, dendritic cells (DCs), monocytes, and neutrophils. It has been reported that MDSCs were involved in tumor‐associated immunosuppression and played immune regulatory roles in infectious disease, autoimmune disease, trauma, and inflammatory diseases 1, 2, 3, 4. MDSCs have abilities to inhibit T‐cell responses involving arginine and cysteine depletion by arginase‐1 (Arg‐1) and the inducible nitric oxidase (iNOS), generation of reactive oxygen species (ROS), expression of heme oxygenase‐1 (HO‐1) 5, 6, 7, 8, 9, 10, 11. In addition, MDSCs regulate immunity through several mechanisms that include attenuating the cytotoxicity of natural killer cells (NK), inducing T regulatory cells (Treg), and polarizing immunity toward a tumor‐promoting type 2 phenotype through downregulation of interferon‐*γ*(IFN‐*γ*) and upregulation of interleukin‐10 (IL‐10), etc. 5, 6, 7, 8. CD14^+^HLA‐DR^‐/low^ MDSCs were one of the few well‐characterized MDSC subsets in human. In the past few years, the immunosuppressive functions of CD14^+^HLA‐DR^‐/low^ MDSCs were described by several researches, and the expansion and functional importance of CD14^+^HLA‐DR^‐/low^ MDSCs in patients with cancer and noncancer pathogenic conditions has been recognized 12, 13, 14, 15, 16, 17, 18, 19, 20, 21, 22, 23, 24, 25, 26, 27, 28. As a result, there has been growing interest in understanding their roles in acute graft‐versus‐host disease (aGVHD) after allogenetic hematopoietic stem cell transplantation (allo‐HSCT) 29, 30, 31. In murine aGVHD transplant models, MDSC accumulation in recipient mice posttransplantation was positively correlated with the severity of aGVHD 32, 33. The infusion of MDSCs before transplantation provided protection from lethal acute GVHD, leading to long‐term survival of recipient mice 32, 33. So far, the relation of MDSC subsets to aGVHD in patients with allo‐HSCT have not been well defined. Therefore, in this study, we not only systematically detected the dynamic changes of MDSCs accumulation in patients during the first 100 days after allo‐HSCT, but also investigated the changes in frequencies of other cell types and concentrations of relative cytokines during the accumulation of MDSCs, with the purpose of evaluating possible roles of CD14^+^HLA‐DR^‐/low^ MDSCs in allo‐HSCT, especially in aGVHD development.

## Materials and Methods

### Patients

This study included 30 leukemia patients undergoing allo‐HSCT in our institution in 2012. This study was approved by the Medical Ethics Committee of the Tongji Hospital. All patients gave written informed consent to sampling of blood and collection of clinical data in accordance with the Declaration of Helsinki. The characteristics of the patients are summarized in Table 1. The median age was 28 years. There were 17 males and 13 females. The primary diseases included acute lymphoblastic leukemia (ALL, *n *=* *8), acute myeloid leukemia (AML, *n *=* *15), chronic myeloid leukemia in accelerated or blast phase (CML, *n *=* *2), high risk myelodysplastic syndrome (MDS, *n *=* *5). Patients received grafts from matched sibling donors (*n *=* *20), mismatched sibling donors (*n *=* *5), and matched unrelated donors (MUD, *n *=* *5). All donors had high‐resolution molecular typing for HLA‐A, ‐B, ‐C, HLA‐DRB1, and DQB1. The donors were mobilized with granulocyte colony‐stimulating factor (G‐CSF, 5 *μ*g/kg/day) for 4–5 days. A minimum of 5.0 × 10^8^/kg MNC and 4.0 × 10^6^/kg CD34^+^ cells in the graft were considered appropriate to safely carry out transplantation. Twenty‐two patients received BuFlu conditioning regimens. Eight patients received BuCy2 conditioning regimens. Standard protocol with cyclosporin A (CsA) plus methotrexate (MTX) were administrated for GVHD prophylaxis 34. MMF, in combination with CsA and MTX, was used in relatives of HLA mismatched transplantation, unrelated donor transplantation. The information on stem cell donors and patients is given in Table 1.

**Table 1:** The characteristic of the patients

Patient characteristics	Values
(a) *The characteristic of the patients and donors with allo‐HSCT*	
Number of patients	30
Number of donors	30
Median patient age, year (range)	28 (14–52)
Median donor age, year (range)	35 (19–50)
Sex, no. (%)	
Male/female patients	17/13 (56.7/43.3)
Male/female donors	18/12 (60/40)
Disease diagnosis, no. (%)	
ALL	8 (26.7)
AML	15 (50)
MDS	5 (16.7)
CML	2 (6.7)
Disease status at transplantation, no. (%)	
CR	29 (96.7)
PR	1 (3.3)
Donor characteristics, no. (%)	
Matched sibling donor	20 (66.7)
Mismatched sibling donor	5 (16.7)
MUD	5 (16.7)
Conditioning regimen	
Ara‐C/Bu/Flu/Me‐CCNU	22 (73.3)
Ara‐C/Bu/Cy/Me‐CCNU	8 (26.7)
GVHD prophylaxis	
CsA+MTX	20 (66.7)
CsA+MTX+MMF	10 (33.3)
Mobilization regimen	
G‐CSF	30 (100)
Stem cell source	
Bone marrow + peripheral blood	3 (10)
Peripheral blood	27 (90)
(b) *The characteristic of the patients developing aGVHD after allo‐HSCT*	
Number	17
Median age, year (range)	24 (14–52)
Sex, no.(%)	
Male	10 (58.8)
Female	7 (41.2)
Disease diagnosis, no. (%)	
ALL	6 (35.3)
AML	8 (47.1)
MDS	2 (11.8)
CML	1 (5.9)
Disease status at transplantation, no. (%)	
CR	16 (94.1)
PR	1 (5.9)
Donor characteristics, no. (%)	
Matched sibling donor	11 (64.7)
Mismatched sibling donor	3 (17.6)
MUD	3 (17.6)
Conditioning regimen	
Ara‐C/Bu/Flu/Me‐CCNU	10 (58.8)
Ara‐C/Bu/Cy/Me‐CCNU	7 (41.2)
GVHD prophylaxis	
CsA+MTX	11 (64.7)
CsA+MTX+MMF	6 (35.3)
Stem cell source	
Bone marrow + peripheral blood	3 (17.6)
Peripheral blood	14 (82.4)

MUD, matched unrelated donor; Ara‐C/Bu/Cy/Me‐CCNU, cytarabine (2–4 g/m^2^/day on days −10, −9), busulfan (3.2 mg/kg/day IV on days −8 to −6), cyclophosphamide (1.8 g/m^2^/day on days −5 and −4), Semustine (250 mg/m^2^/day on days −3); Ara‐C/Bu/Flu/Me‐CCNU, cytarabine (2–4 g/m^2^/day on days −10, −9), busulfan (3.2 mg/kg/day IV on days −7 to −5), fludarabine (30 mg/m^2^/day on days −12 to −8), Semustine (250 mg/m^2^/day on days −3); CsA, cyclosporin A (2.5 mg/kg/day every 12 h since day −10); MTX, methotrexate (15 mg/m^2^ on day 1 and 10 mg/m^2^ on days +3, +6, and +11); MMF, mycophenolate mofetil (1.0 g/day, from the beginning of conditioning therapy); G‐CSF, granulocyte colony‐stimulating factor. The donors were mobilized with G‐CSF (5 *μ*g/kg/day) for 4–5 days.

Peripheral blood was obtained from all patients at time points of engraftment, 28, 35, 49, 63, 77, and 91 day after allo‐HSCT. For patients developing aGVHD, peripheral blood was collected when aGVHD occurred and after aGVHD treated, respectively. For healthy controls, peripheral blood was collected from volunteers. All the analyses were performed on freshly harvested cells.

### Antibodies and flow cytometric analysis

The following fluorescein isothiocyanate (FITC), phycoerythrin (PE), Peridinin chlorophyII protein (PerCP), allophycocyanin (APC) conjugated mAbs were utilized: CD45‐PerCP, CD4‐FITC, CD14‐FITC, CD3‐PE, HLA‐DR‐PE, CD8‐APC, CD25‐APC, CD33‐APC, CD123‐APC, Lineage‐FITC, CD11c‐PE, Foxp3‐PE, HLA‐DR PerCP (Table S1). By multiple immunostaining, we characterized CD3^+^CD4^+^ T helper cells, CD3^+^CD8^+^ cytotoxic T cells, CD4^+^CD25^+^Foxp3^+^ Tregs, CD3^‐^CD56^+^ NK cells. DCs were enumerated with Lineage, HLA‐DR, CD11c, and CD123. MDSCs were stained for the surface marker HLA‐DR, CD33, CD14. Cell were stained with the relevant mAbs and analyzed on a Becton Dickinson (BD, NY, USA) FACS Calibur. Data were analyzed with Cell Quest software (BD Biosciences). The frequencies of relevant cell subsets were calculated among total peripheral blood mononuclear cell (PBMC). For each blood sample, isotype controls and CD45 staining were used to correct for background staining and nonleukocytes.

### Cell isolation

Peripheral blood mononuclear cells were isolated from peripheral blood both of patients and healthy donors using Ficoll separation liquid. CD14^+^HLA‐DR^‐/low^ cells were isolated by magnetic beads separation (Miltenyi Biotec, Bergisch Gladbach, Germany) according to the manufacturer's instructions.

### Cytokine analysis

For measuring the serum concentrations of IL‐6, tumor necrosis factor *α* (TNF‐*α*) (R&D Systems, Minneapolis, MN), Arg‐1, HO‐1, iNOS (Cloud‐clone Corp, Houston, TX) , IL‐10, IL‐1*β* (Biolegend, San Diego, CA), samples were analyzed using ELISA kits following the manufacturer's instructions.

### Statistical analysis

Differences in means and correlation analyses were evaluated with parametric (two‐tailed student or paired *t*‐test and Pearson test) or nonparametric (Mann–Whitney U or Wilcoxon and Spearman *ρ* test) tests based on the distribution levels. All statistical analyses were performed using GraphPad Prism Version 5 (GraphPad Prism Software Inc., San Diego, CA) and SPSS 19.0 software package (SPSS Inc., Chicago, IL) at a significance level of *P *≤* *0.05.

## Results

### Characterization of CD14^+^HLA‐DR^‐/low^ MDSCs in patients with allo‐HSCT

**Figure 1:**
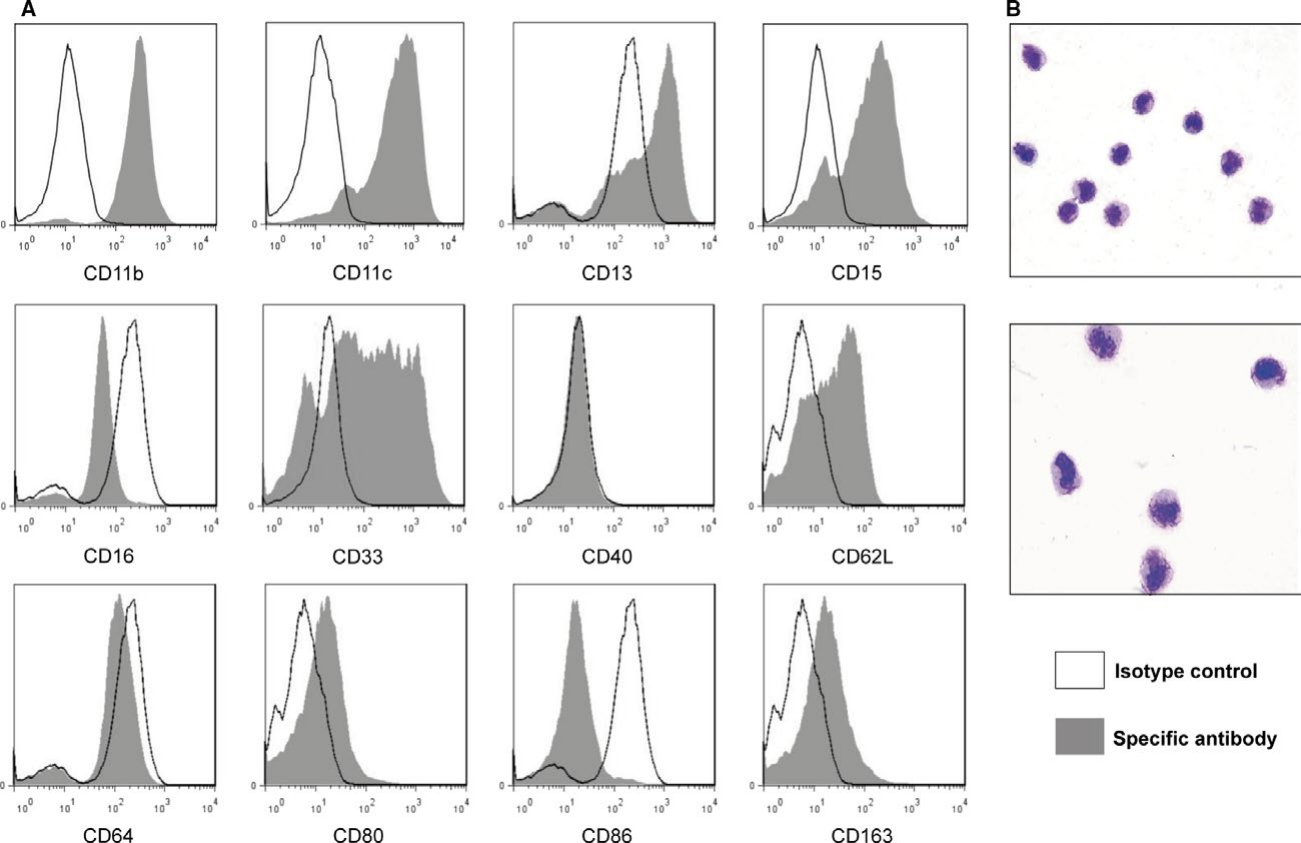
The characteristic of CD14^+^ HLA‐DR ^−/low^ MDSCs. (A) Representative histograms of the phenotypic analyses of CD14^+^ HLA‐DR ^−/low^ MDSCs from patients with allo‐HSCT. (B) The morphology of CD14^+^ HLA‐DR ^−/low^ MDSCs by May‐Grunwald–Giemsa staining.

To identify the phenotype, CD14^+^HLA‐DR^‐/low^ MDSCs were purified from PBMCs of patients. Purity of CD14^+^HLA‐DR^‐/low^ cells from patients was 85–90%. Gating strategy for MDSCs analysis is shown in Figure S1. Representative histograms of the expression of surface marker on MDSCs are shown in Figure 1A. The cells expressed myeloid markers (CD11c, CD33), immature markers (CD15, CD13) and adhesion molecules (CD11b, CD62L). Markers for mature myeloid cells (CD64, CD16) were negative. The scavenger receptor CD163 was positive. Costimulatory molecules CD86 and CD40 were found negative, whereas CD80 were positive. Based on these cell surface markers, Figure 1B shows the morphology of this subset by May‐Grunwald–Giemsa staining. Among MDSCs, those expressing CD14 are usually called monocytic MDSC (M‐MDSCs) by contrast to granulocytic (CD14^−^, CD15^+^) MDSCs (G‐MDSCs). Therefore, the subset analyzed in this study should be identified as M‐MDSCs.

### CD14^+^HLA‐DR^‐/low^ MDSCs frequencies in the graft

**Figure 2:**
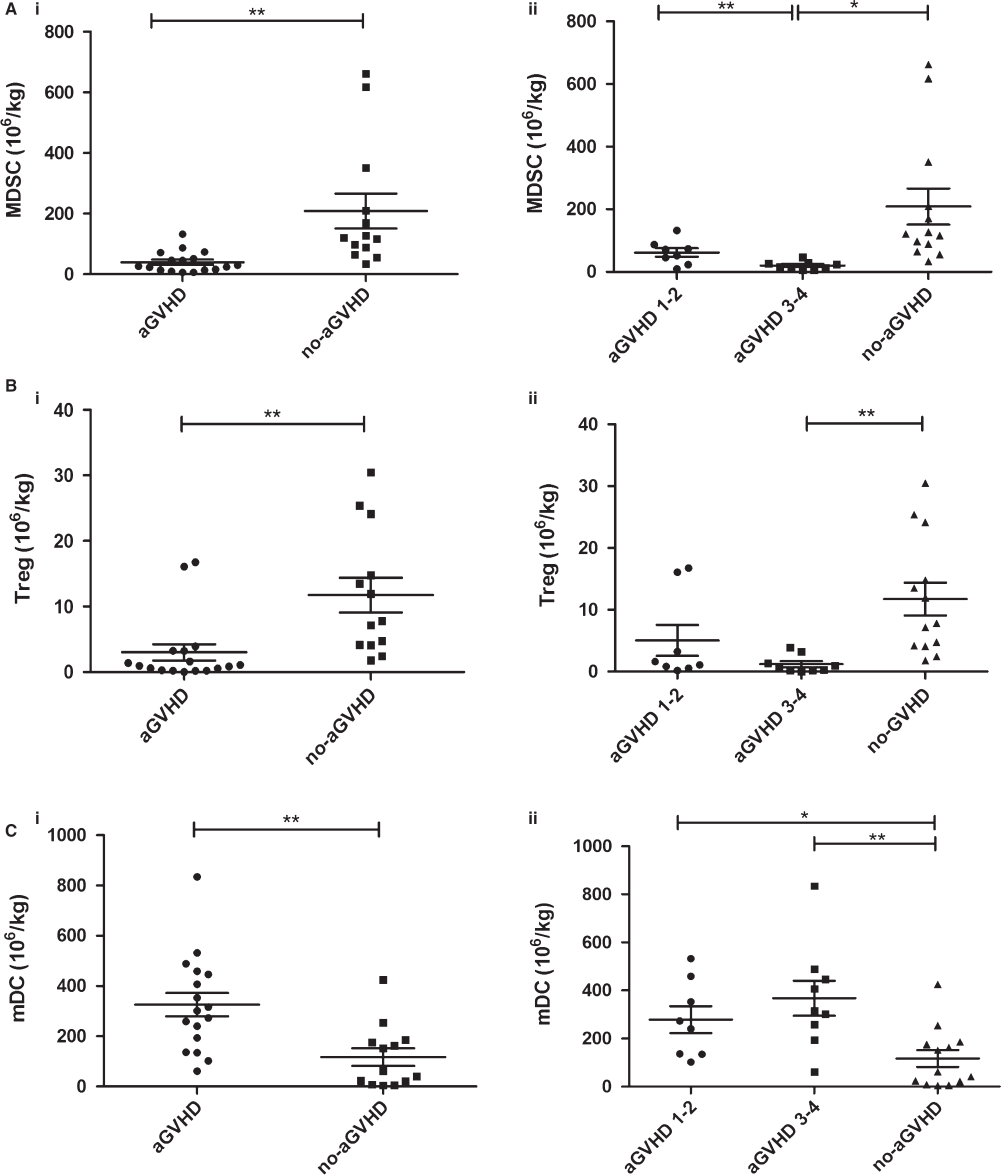
Frequencies of cell subsets in the graft. (A) Frequencies of MDSCs in the graft were compared between patients developing aGVHD and not developing aGVHD (i) and aGVHD scores (ii). (B) Frequencies of Tregs in the graft were compared between developing aGVHD and not developing aGVHD (i) and aGVHD scores (ii). (C) Frequencies of mDCs in the graft were compared between developing aGVHD and not developing aGVHD (i) and aGVHD scores (ii).

Patients developing aGVHD received a mean dose of 39.94 × 10^6^ M‐MDSCs/kg body weight, whereas patients not developing aGVHD received a mean dose of 209.0 × 10^6^ M‐MDSCs/kg body weight. The comparison analysis demonstrated that higher doses of M‐MDSCs in the graft were associated with a minor risk of developing aGVHD (*P *=* *0.0026, Fig. 2Ai). In further analysis, a significant correlation between the number of M‐MDSCs infused at the time of transplantation and the severity of aGVHD was found (Fig. 2Aii). Patients developing aGVHD 1‐2 and patients without aGVHD received mean dose of 61.96 × 10^6^ and 209.0 × 10^6 ^M‐MDSCs/kg body weight, respectively, which were significantly higher than a mean dose of 20.37 × 10^6^ cells/kg body weight in patients developing aGVHD 3‐4. The statistical details are given in Table S2.

### Changes of other cell subsets in the graft

The graft content of different cell population shown to impact on aGVHD was analyzed. No significant correlations were found between the numbers of CD34^+^ cells, CD3^+^ T cells, CD3^+^CD4^+^T helper cells, CD3^+^CD8^+^ cytotoxic T cells, CD3^‐^CD56^+^ NK cells and pDC infused and aGVHD development (Figure S2; Table S2). However, there was a significant association between the number of Tregs infused and aGVHD. Patients developing aGVHD were infused with lower number of Tregs compared with patients not developing aGVHD (mean 3.007 × 10^6^ cells/kg body weight vs. mean 11.73 × 10^6^ cells/kg body weight, *P *=* *0.0033) (Fig. 2Bi). In addition, patients developing aGVHD received higher number of myeloid DCs (mDCs) than patients without aGVHD (mean 325.8 × 10^6^ cells/kg body weight vs. mean 117.0 × 10^6 ^cells/kg body weight, *P *=* *0.0021) (Fig. 2Ci). But further analysis failed to show a significant correlation between the numbers of Tregs and mDCs infused at the time of transplantation and the severity of aGVHD (Fig. 2Bii, 2Cii). The absolute doses of the graft cell populations infused to patients at the time of transplantation are shown in Table S2.

### CD14^+^HLA‐DR^‐/low^ MDSCs levels at the time of engraftment

**Figure 3:**
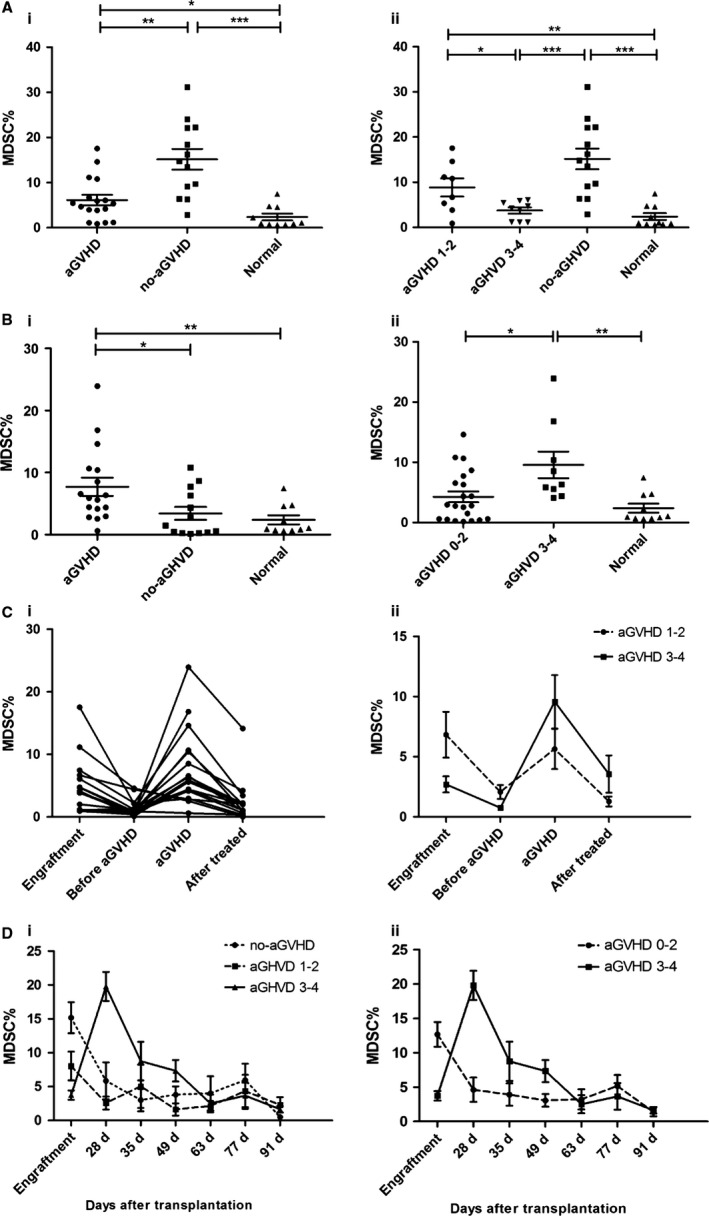
Increased proportion of MDSCs in PBMCs of patients after allo‐HSCT. (A) At the time of engraftment, the levels of MDSCs in PBMCs were compared between patients and normal controls (i), and were further analyzed according to aGVHD scores (ii). (B) After allo‐HSCT, comparisons of MDSCs frequencies were performed between patients and normal controls grouped by aGVHD (i) and aGVHD severity (ii). (C) The dynamic changes of MDSCs frequencies after allo‐HSCT were monitored in patients with aGVHD (i) and were analyzed based on aGVHD scores (ii). The systematic monitoring of MDSCs frequencies was performed in all patients during the first 100 days after allo‐HSCT grouped by aGVHD scores (iii) and aGVHD severity (iv).

In patients undergoing allo‐HSCT, the mean percentage of M‐MDSCs at the time of engraftment was higher than that in normal controls (mean 2.392 ± 0.756%) (Fig. 3Ai). At the time of engraftment, the mean percentage of M‐MDSCs in patients not developing aGVHD was significantly higher than that in patients developing aGVHD (15.16 ± 2.294% vs. 6.148 ± 1.165%, *P = *0.0017). What's more, a significant correlation between the levels of M‐MDSCs at the time of engraftment and the severity of aGVHD was found (Fig. 3Aii). But, the levels of M‐MDSCs had no significant difference between patients with aGVHD 1‐2 and without aGVHD, neither patients with aGVHD 3‐4 or normal controls. The results are listed in Table S3a. These findings are in line with the results of analysis on M‐MDSCs numbers in the graft, which found a significant association between higher M‐MDSCs levels and lower risks of developing aGVHD.

### Increased proportion of CD14^+^HLA‐DR^‐/low^ MDSCs at aGVHD onset

In this study, aGVHD was documented in 17 patients (56.67%), of whom nine had grade 3‐4 disease (30%). Thirteen patients (43.33%) without aGVHD were observed. After allo‐HSCT, the mean percentage of M‐MDSCs increased markedly in patients developing aGVHD compared with patients not developing aGVHD and normal controls (7.725 ± 1.460% vs. 3.423 ± 1.044%, *P *=* *0.0213; 7.725 ± 1.460% vs. 2.392 ± 0.756%, *P *=* *0.0084). No difference in M‐MDSCs frequencies between patients without aGVHD and normal controls was observed (*P *=* *0.7802) (Fig. 3Bi). Grouped by aGVHD scores, it showed that patients with severe aGVHD (grade 3‐4) had significantly higher proportion of M‐MDSCs than patients with no or mild aGVHD (grade 0‐2) (Fig. 3Bii). The statistical details are shown in Table S3b.

And according to our continuous monitoring on M‐MDSCs levels in patients developing aGVHD, the proportion of M‐MDSCs decreased from a higher level at the time of engraftment to a lower level before aGVHD occurred. After aGVHD occurred, the level of M‐MDSCs increased again and a synchronized reduction in the level of M‐MDSCs was observed in patients with the alleviation of aGVHD after effective management with immunosuppressive therapy (Fig. 3Ci, ii). An overview of the dynamic changes of M‐MDSCs frequencies in all patients during the first 100 days after allo‐HSCT is shown in Figure 3Ciii, 3Civ.

### Changes of other cell subsets during CD14^+^HLA‐DR^‐/low^ MDSCs accumulation after allo‐HSCT

**Figure 4:**
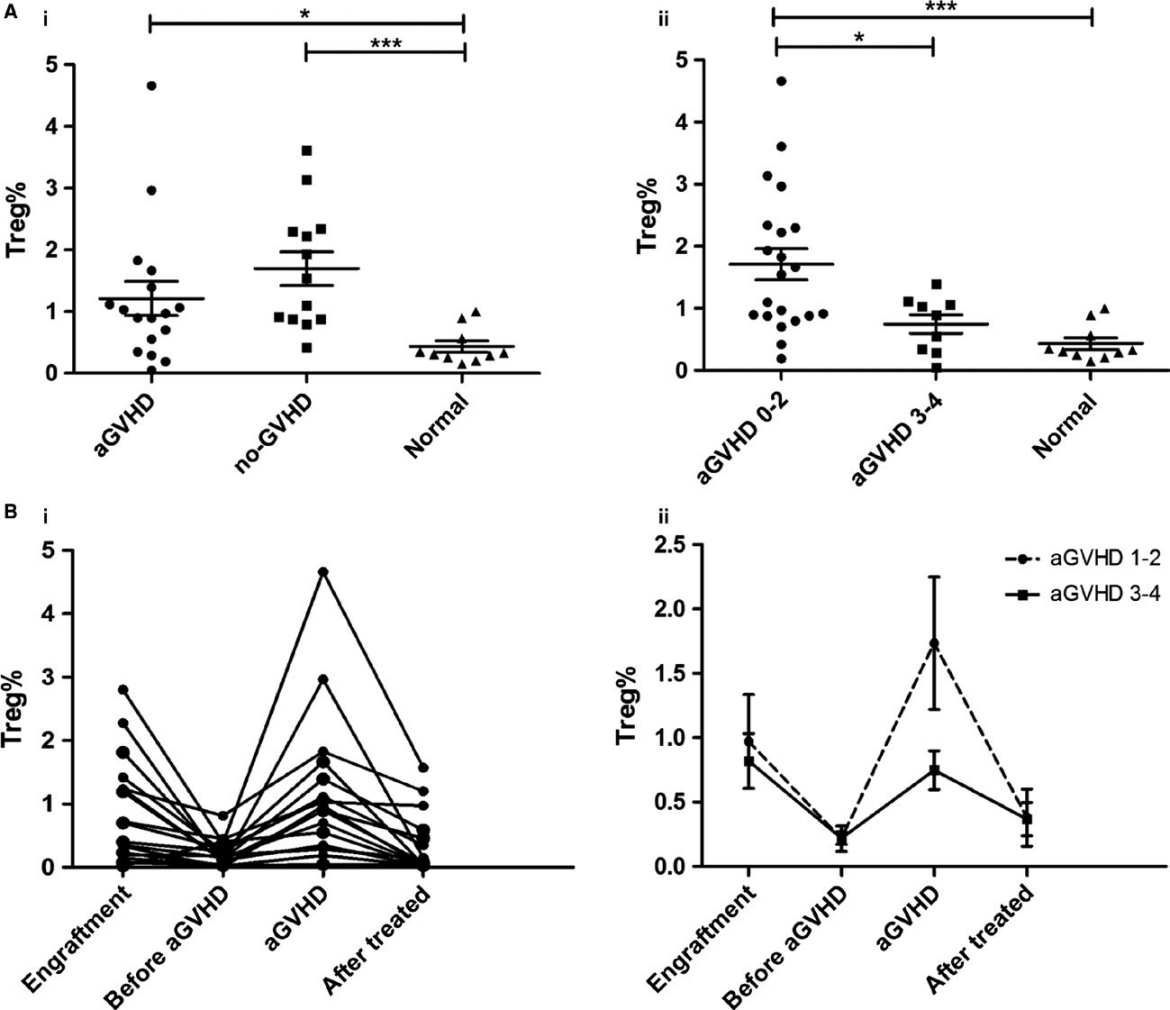
Changes of cell subsets in PBMCs of patients after allo‐HSCT. (A) The frequencies of Tregs after allo‐HSCT were compared between patients and normal controls grouped by aGVHD (i) and aGVHD severity (ii). (B) The dynamic changes of Tregs frequencies after allo‐HSCT were monitored in patients with aGVHD (i) and were analyzed based on aGVHD scores (ii).

No difference was found in the levels of Tregs between patients with aGVHD and no‐aGVHD after allo‐HSCT (1.212 ± 0.275% vs. 1.697 ± 0.273%, *P *=* *0.230). But the proportion of Tregs was detected significantly higher in patients with no or mild aGVHD (0‐2) than in patients with severe aGVHD (3‐4) and normal controls (1.711 ± 0.251% vs. 0.748 ± 0.151%, *P *=* *0.0374; 1.711 ± 0.251% vs. 0.434 ± 0.092%, *P *=* *0.0006). And there was no statistical difference in the levels of Tregs between patients with aGVHD 3‐4 and normal controls (*P *=* *0.1023) (Fig. 4A). In this study, the dynamic changes of the levels of Tregs in patients developing aGVHD were similar to that of M‐MDSCs (Fig. 4Bi). The percentages of Tregs decreased from higher levels at the time of engraftment to lower levels before aGVHD occurred. The levels obviously increased again at aGVHD onset and decreased after effective management with immunosuppressive therapy (Fig. 4Bii). Besides, no differences were observed in the mean percentage of mDCs and pDCs after allo‐HSCT between patients developing aGVHD and patients not developing aGHVD (data not show).

### CD14^+^HLA‐DR^−/low^ MDSCs in relation to other cell subsets

**Figure 5:**
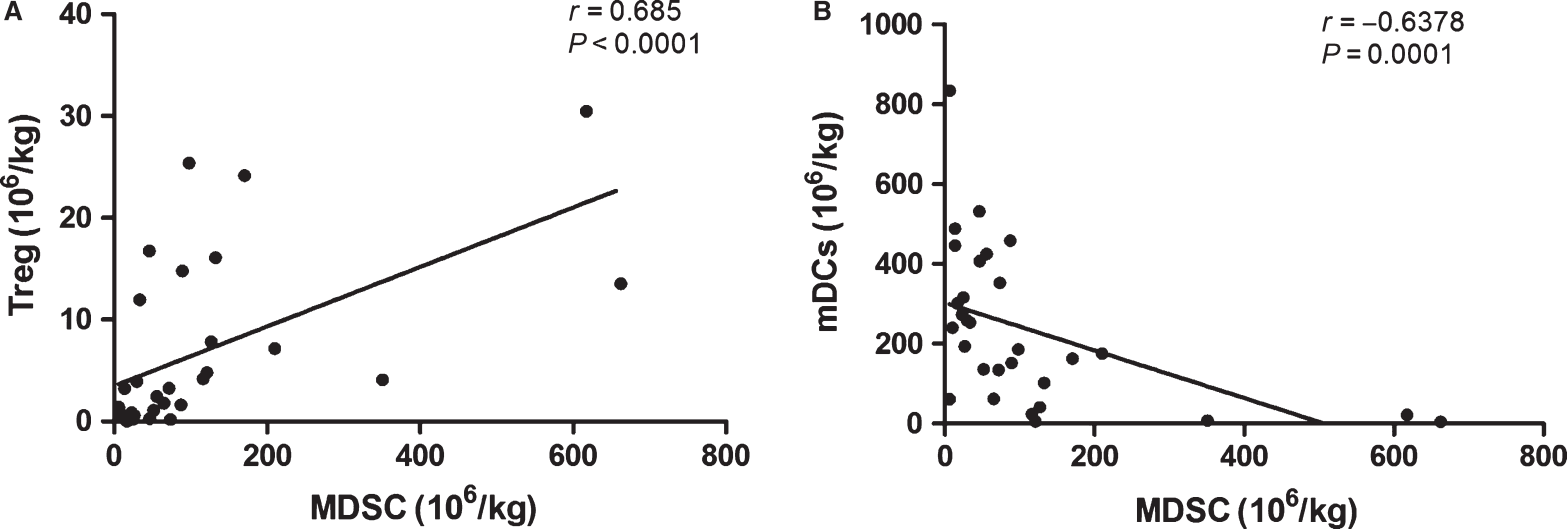
Correlations between the frequencies of MDSCs and other PBMCs subsets in the graft. (A) Correlations between the frequencies of MDSCs and Tregs in the graft. (B) Correlation between the frequencies of MDSCs and mDCs in the graft.

As MDSCs consists of immature myeloid cells and the progenitor cells, accumulation of MDSCs may affect the number of other PBMCs subsets. Therefore, we assessed the relative subsets in the graft and in PBMCs of patients after allo‐HSCT. In the graft, a positive correlation between M‐MDSCs and Tregs, and a negative correlation between M‐MDSCs and mDCs were found (Fig. 5). However, correlations were observed neither between M‐MDSCs and Tregs nor between M‐MDSCs and mDCs in PBMCs after allo‐HSCT. In addition, T cells and pDCs did not show obvious correlations with M‐MDSCs both in the graft and in PBMCs after allo‐HSCT (Figure S3).

### Survival analysis

**Figure 6:**
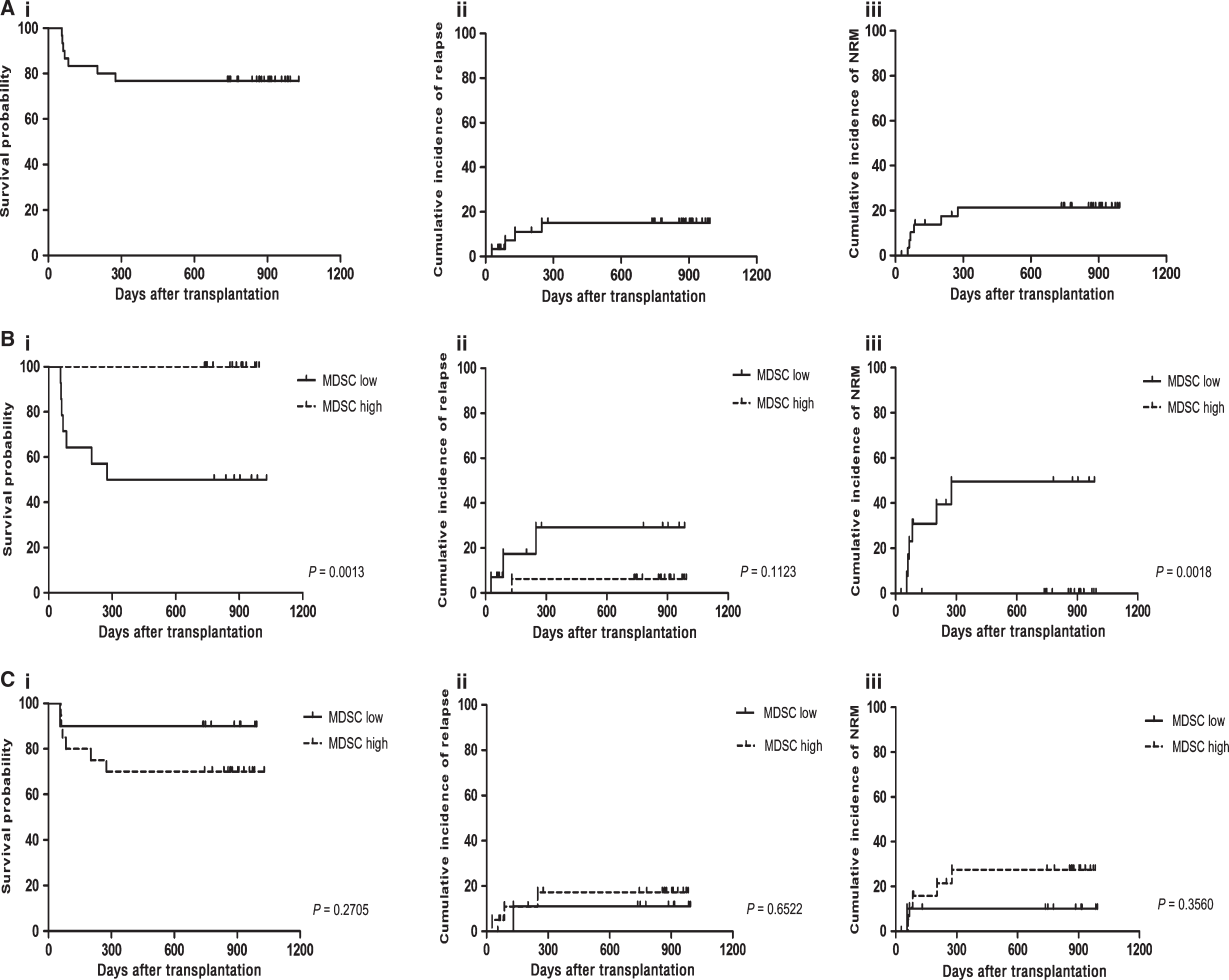
Clinical outcomes of the patients with allo‐HSCT. (A) Survival analyses were performed in patients in terms of overall survival (i), cumulative incidence of relapse (ii), and cumulative incidence of NRM (iii). (B) Overall survival (i), cumulative incidence of relapse (ii), and cumulative incidence of NRM (iii) of the patients were analyzed grouped by MDSCs levels in the graft. (C) Overall survival (i), cumulative incidence of relapse (ii), and cumulative incidence of NRM (iii) of the patients were analyzed grouped by MDSCs levels in PBMCs after allo‐HSCT.

With a median follow‐up of 701 days (range 27–1014 days), 2‐year overall survival (OS) was 76.67% (Fig. 6Ai). The cumulative incidence of relapse for all patients at 100 days and 2 years were 7.20% and 15.11% (Fig. 6Aii). And the cumulative incidence of nonrelapse mortality (NRM) at 2 years was 21.47% (Fig. 6Aiii). At the end of follow‐up, 23 patients are alive including three patients who relapsed at the time of 2.9 months, 4.3 months, and 8.3 months after transplantation. One patient died because of disease relapse. One patient died of viral myocarditis. Two patients died from severe pneumonia. Three deaths were related to grade 4 aGVHD.

**Figure 7:**
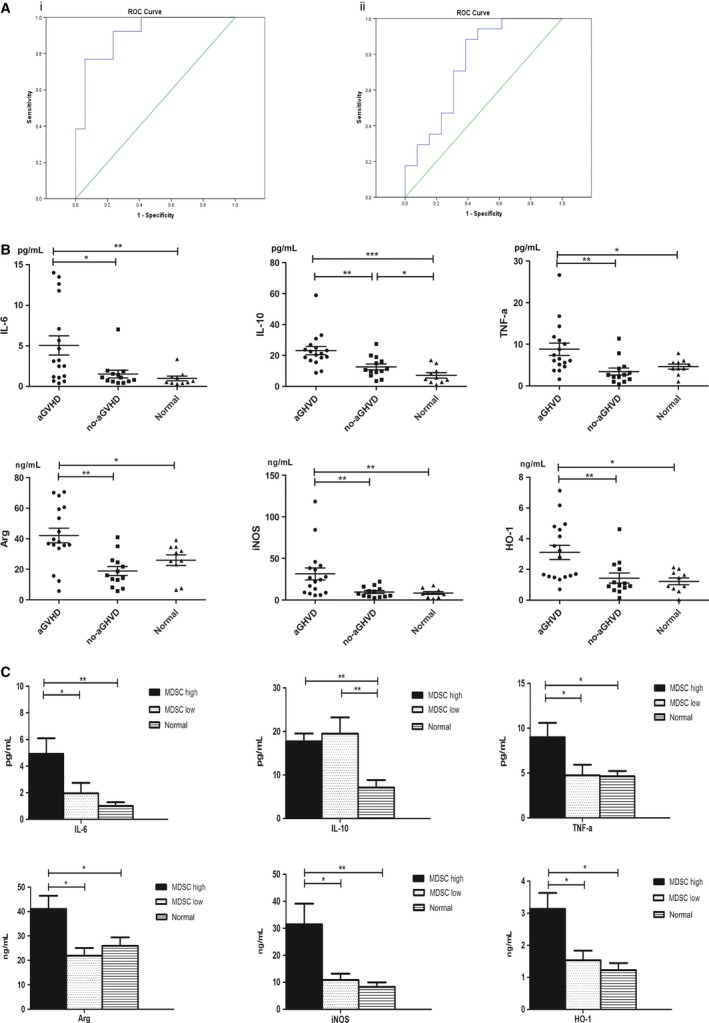
Concentrations of cytokines in patients after allo‐HSCT. (A) ROC analysis of MDSCs numbers in the graft (i) and ROC analysis of MDSCs proportions in PBMCs after allo‐HSCT (ii). (B) Concentrations of cytokines in patients after allo‐HSCT. (C) Concentration of cytokines in patients grouped by MDSCs levels after allo‐HSCT.

In order to further investigate its influences on clinical outcomes, we performed ROC analyses of M‐MDSCs levels in the graft and in PBMCs after allo‐HSCT. According to the ROC analysis on M‐MDSCs number in graft, the area under the ROC curve for M‐MDSCs numbers in the graft was 0.91 (95% confidence interval (CI), 0.806–1.000; *P *<* *0.0001), setting the optimal cut‐off value at 53.712 × 10^6^ cells/kg body weight, gave a sensitivity of 0.923 and a specificity of 0.765 on the ROC curve (Fig. 7Ai). The high MDSCs group (>53.712 × 10^6 ^M‐MDSCs/kg body weight) showed more favorable clinical outcomes compared with the low MDSCs group (≤53.712 × 10^6 ^M‐MDSCs/kg body weight). The 2‐year OS were 100% in high MDSCs group compared with 50% in low MDSCs group (*P *=* *0.0013, Fig. 6Bi). The cumulative incidence of relapse at 2 years were 6.250% and 29.252% in high MDSCs group and low MDSCs group, respectively (*P *=* *0.1123, Fig. 6Bii). The cumulative incidence of NRM was significantly lower in high MDSCs group (0%) in comparison with low MDSCs group (49.519%, *P *=* *0.0018, Fig. 6Biii).

ROC analysis showed that the area under the ROC curve for M‐MDSCs levels after allo‐HSCT was 0.751 (95% confidence interval (CI), 0.565 to 0.937; *P *=* *0.02), setting the optimal cut‐off value at 2.798% gave a sensitivity of 0.882 and a specificity of 0.615 on the ROC curve (Fig. 7Aii). After allo‐HSCT, patients in high MDSCs group (>2.798%) seemed to have a poor prognosis compared with patients in low MDSCs group (≤2.798%). But no statistical differences were observed. The 2‐year OS were 70% in high MDSCs group and 90% in low MDSCs group (*P *=* *0.2705, Fig. 6Ci). The cumulative incidence of relapse at 2 years were 17.299% in high MDSCs group and 11.11% in low MDSCs group (*P *=* *0.6522, Fig. 6Cii). And the 2‐year cumulative incidence of NRM were 27.449% in high MDSCs group and 10% in low MDSCs group, respectively (*P *=* *0.3560, Fig. 6Ciii). All the details are shown in Table S4.

### Relations between CD14^+^HLA‐DR^‐/low^ MDSCs, cytokines, and aGVHD

Several cytokines correlated with the accumulation, differentiation, activation of the MDSCs (IL‐6, IL‐10, IL‐I*β*, TNF‐*α*) and the immunosuppressive functions of MDSCs (Arg‐1, iNOS, HO‐1) were detected in our study. After allo‐HSCT, the concentrations of IL‐6, IL‐10, TNF‐*α*, Arg‐1, iNOS, and HO‐1) were significant elevated in patients developing aGVHD (Fig. 7B). Moreover, the significant differences in IL‐6, TNF‐*α*, Arg‐1, iNOS, and HO‐1 levels were also observed between patients with MDSCs proportion >2.798% and patients with MDSCs proportion ≤2.798% (Fig. 7C). Although patients after allo‐HSCT had higher level of IL‐10 compared with normal controls, no difference was found between patients with MDSCs proportion >2.798% and patients with MDSCs proportion ≤2.798% (Fig. 7C). There was no difference in IL‐1*β* levels between patients with aGVHD and no‐aGVHD (data not shown). The mean concentrations of the cytokines involved are listed in Table S5.

## Discussion

In humans, MDSCs commonly express myeloid marker CD11b and/or CD33 and lack or weakly express HLA‐DR. Although CD14^+^HLA‐DR^‐/low^ MDSCs is one of the few well‐characterized MDSC subsets in human, the markers of MDSCs are still being debated owing to the lack of specific markers 4, 16, 17, 21, 25, 29, 35, 36, 37. Thus, we purified CD14^+^HLA‐DR^‐/low^ MDSCs from PBMCs of our patients to identify the phenotype. It is found that MDSC subset analyzed in this study should be identified as M‐MDSCs by contrast to G‐MDSCs.

Although several studies on solid organ transplantation reported that MDSCs have been associated with better tolerance and long‐term survival, the studies about MDSCs in allo‐HSCT have been limited 12, 13, 14, 15, 29, 30, 31. The latest investigations found that the changes of MDSCs frequencies had links to occurrence of aGVHD and the number of MDSCs infused did not impact the relapse rate or the transplant‐related mortality rate 29, 30, 31. Our finding is further evidence that higher number of M‐MDSCs in the graft would apparently reduce risks of aGVHD. Furthermore, a significant correlation between the number of M‐MDSCs infused and the severity of aGVHD was found in our study. Lower the numbers of M‐MDSCs infused were, the severer the aGVHD after allo‐HSCT would be. On the basis of our findings above, we performed ROC analyses of M‐MDSCs levels in the graft to identify a dose of infused M‐MDSCs able to exert a protective effect on aGVHD. Results showed that the graft dose of 53.712 × 10^6^ MDSCs/kg body weight is able to discriminate patients developing aGVHD after allo‐HSCT with a specificity of 76.5% and a sensitivity of 92.3%. Patients received a dose of greater than 53.712 × 10^6^ MDSCs/kg body weight had significantly better 2‐year OS and the cumulative incidence of NRM. And the cumulative incidence of relapse at 2 years was not influenced by the high level of M‐MDSCs in the graft. These findings were in line with that reported by Vendramin A and Lv M et al. very recently 30, 31. Both of the studies demonstrated the positive effects of high levels of MDSCs in the graft. Furthermore, even when donor characteristics (age, sex, and graft content) were taken into account, multivariate analysis confirmed that the number of M‐MDSC/kg of body weight is the only independent factor associated with the occurrence of aGVHD (Table S6). Therefore, we speculate that if the number of M‐MDSCs infused is greater than 53.712 × 10^6^ MDSCs/kg body weight, patients will have lower risks of aGVHD and consequently have favorable clinical outcomes. But if patients receive the graft with M‐MDSCs number lower than 53.712 × 10^6^ MDSCs/kg body weight, they will be more likely to develop aGVHD. The lower the M‐MDSCs number is, the severer the aGVHD will be. And poor prognosis will be inevitable. Thus, patients received low number of MDSCs should be closely observed and treated timely during the treatment.

Although previous studies provided proof that M‐MDSCs in the graft had positive effects on aGVHD development and clinical outcomes, none of them comprehensively monitored the changes of M‐MDSCs frequencies since the day of transplantation till the 100 days after allo‐HSCT 29, 30, 31. In this study, we systematically and continuously monitored the changes of M‐MDSCs frequencies in order to give a full assessment of effects of M‐MDSCs on patients undergoing allo‐HSCT. In patients with mild aGVHD (0‐2), M‐MDSCs accumulated at the time of engraftment after allo‐HSCT and decreased to basal levels at about 4 weeks. M‐MDSCs frequencies would keep in stable levels with slight fluctuations in the following weeks. But, in patients with severe aGVHD (3‐4), M‐MDSCs elevated slightly when engraftment. When aGVHD occurred, M‐MDSCs frequencies would significantly increase. And a synchronized reduction in the levels of M‐MDSCs was observed after effective management of aGVHD with immunosuppressive therapy. Given these findings, we performed further analyses with the purpose of determining whether M‐MDSCs accumulations at the time of engraftment and at aGVHD onset were related to prognosis in patients undergoing allo‐HSCT. Results showed that, at the time of engraftment, M‐MDSCs level in PBMCs had a prognostic ability similar to the number of M‐MDSCs in the graft. The higher the proportion of MDSCs in peripheral blood at the time of engraftment, the lower the risk of aGVHD occurrence. However, the level of M‐MDSCs when engraftment could be used only to indicate the possibility of occurrence of severe aGVHD (3‐4), not to be able to distinguish patients developing aGVHD 1‐2 from no‐aGVHD (Figure S4). Thus, we came to the conclusion that the number of M‐MDSCs in the graft had more favorable predictive abilities compared with that in PBMCs when engraftment after transplantation.

But on the contrary, significantly raised M‐MDSCs frequencies were detected when patients developed aGVHD. The severer aGVHD was, the higher M‐MDSCs frequencies would be. Nevertheless, the M‐MDSCs accumulation when aGHVD occurred had no influences on OS, the cumulative incidence of NRM and relapse. These results were consistent with previous studies 31. In vitro experiments showed that the expansion and activation of MDSCs were greatly regulated by inflammatory factors such as TNF‐*α*, TGF‐*β*, IL‐6, IL‐1*β*, IL‐10, vascular endothelial growth factor (VEGF), prostaglandin E2 (PGE2), granulocyte‐macrophage colony‐stimulating factor (GM‐CSF), and macrophage colony‐stimulating factor (M‐CSF) 1, 4, 38, 39. In order to confirm whether the accumulation of MDSCs in patients after allo‐HSCT is related to the change in concentrations of inflammatory factors, we detected the levels of IL‐10, IL‐6, IL‐*β*, and TNF‐*α* relevant to the expansion of MDSCs in patients after allo‐HSCT 1, 4, 38, 39, 40, 41, 42, 43, 44, 45, 46, 47. Concentrations of IL‐10, IL‐6, and TNF‐*α* were significantly higher in patients developing aGVHD than that in patients without aGVHD. We further analyzed the concentrations of cytokines according to the result of ROC analysis of M‐MDSCs levels after allo‐HSCT. Concentrations of IL‐6 and TNF‐*α* increased greatly in high MDSCs group (>2.798%) compared with low MDSCs group (<2.798%). In addition, we also detected the levels of Arg‐1, iNOS, and HO‐1 in patients, which were reported to be involved in the suppressive activity of MDSCs. The suppressive activity of MDSCs has been associated with the metabolism of L‐arginine. L‐arginine serves as a substrate for two enzymes, iNOS and Arg‐1. In vitro experiments reported that MDSCs expressed high levels of Arg‐1 and iNOS. And a direct role for these enzymes in the inhibition of T‐cell function was well established 33, 48, 49. Besides, HO‐1‐dependent MDSCs‐mediated alloreactive T‐cell suppression was also reported 50. HO‐1 catabolizes pro‐oxidant heme groups into carbon monoxide, biliverdin, and ferritin, three metabolites involved in immunoregulatory processes 10, 11. In our study, elevated concentrations of Arg‐1, iNOS, and HO‐1 were detected in patients developing aGVHD compared with patients not developing aGVHD. And Arg‐1, iNOS, and HO‐1 levels were also significantly higher in high MDSCs group than that in low MDSCs group.

Combined with our results above, we speculated that the initial accumulation of M‐MDSCs at the time of engraftment after allo‐HSCT mainly resulted from hematopoietic reconstruction. Moreover, conditioning regimens for allo‐HSCT would lead to tissue damage and subsequent inflammation. The inflammatory cytokines release might also cause the accumulation of M‐MDSCs when engraftment. After allo‐HSCT, alloreactive donor T cells would attack healthy tissue of the host resulting in a highly inflammatory condition known as GVHD. As a result, high levels of inflammatory cytokines, especially IL‐6 and TNF‐*α*, released and promoted the expansion of M‐MDSCs in patients developing aGVHD. Consequently, the induced M‐MDSCs probably released high concentrations of Arg‐1, iNOS, and HO‐1 after contacted with activated effector T cells and specifically controlled their proliferative response during aGVHD period. However, although M‐MDSCs strongly accumulated and expressed high levels of Arg‐1, iNOS, and HO‐1, these physiological reactions for the control of inflammatory damage may not be able to counterbalance the induction and aggravation of aGVHD by proinflammatory factors. Therefore, we considered the expansion of M‐MDSCs at aGVHD onset after allo‐HSCT as a secondary inflammatory response. It might be part of a potential negative feedback pathway corresponding to the pathogenesis of aGVHD.

Host myeloid‐derived cells, such as DCs acting as antigen‐presenting cells, are important mediators for the initiation of GVHD. In some studies on cancer patients, accumulation of MDSCs was linked to the impairment of development and function of DCs 51, 52. In our study, higher mDCs content in the graft correlated with severer aGVHD. And the number of mDCs correlated negatively with the number of M‐MDSCs in the graft. Since MDSCs and DCs share a common progenitor cell and mDCs are identified as the dominant type of DCs, the reduction in mDCs levels may be caused by the skewing of the common MDSCs/DC progenitor towards the preferential differentiation of MDSCs at the expense of DCs. Moreover, we found that the changing trend of the levels of Tregs was similar to that of M‐MDSCs both in the graft and in PBMCs after allo‐HSCT. These results were consistent with the findings of Magenau et al. 53. Frequencies of Tregs decreased linearly with increasing grades of aGVHD and the proportion of Tregs at onset of aGVHD predicted the response to aGVHD treatment 53. Although some studies mentioned that MDSCs could promote the activation and expansion of Tregs, no correlation was observed in our study 16, 54, 55, 56, 57. Furthermore, some studies demonstrated that MDSCs induced antigen‐specific tolerance of CD8^+^ and/or CD4^+^ T cells in mice models 9, 56, 58, 59, 60, 61, 62. Neither CD4^+^ T cell nor CD8^+^ T cell was found to have correlation with MDSCs in our study. And in contrast with recent reports, our study analysis failed to reveal a correlation between either the total NK population or the T cells and aGHVD 64 But this is coincident with the results reported by Mougiakakos et al. 29. It may be speculated that the complexity of the microenvironment after allo‐HSCT with respect to the milieu of cytokines and chemokines might explains these differences.

## Conclusion

In patients undergoing allo‐HSCT, immunosuppressive strategies to control aGVHD are only partially effective and tolerance induction is a highly desirable goal in the transplantation. Although our data are determined in a relatively small cohort and that further analyses in prospective studies are required, we suggested that MDSC‐based approaches might constitute a potential new therapeutic option for aGVHD and achieve long‐term immunological tolerance and survival. Several studies in mice models have reported that adoptive transfer of in vitro generated MDSCs can effectively prevent lethal aGVHD, leading to long‐term survival of treated mice, but further investigations of the mechanisms of MDSCs in patients are necessary 61, 63. Further understanding of the activation and differentiation of MDSCs in human will help to develop inhibitors during their unwanted activity, such as preventing relapse, but also provide options for therapeutical intervention in aGVHD after allo‐HSCT.

## Conflict of Interest

All authors declare no conflicts of interest.

## Supporting information

Figure S1. Gating strategy for MDSCs analysis. (A) Flow cytometric analysis of MDSCs in PBMCs of patients. (B) Flow cytometric analysis of MDSCs after purification.

Figure S2. The graft content of different cell population shown to impact on aGVHD was analyzed. No significant correlations were found between the numbers of CD34^+^ cells, CD3^+^ T cells, CD3^+^CD4^+^T helper cells, CD3^+^CD8^+^ cytotoxic T cells, CD3^−^CD56^+^ NK cells, and pDC infused and aGVHD development.

Figure S3. Correlation between the frequencies of MDSCs and other PBMCs subsets. (A) Correlations between the frequencies of MDSCs and other PBMCs subsets in the graft. (B) Correlations between the frequencies of MDSCs and other PBMCs subsets after allo‐HSCT.

Figure S4. MDSCs frequencies at the time of engraftment. Since no difference was observed between patients with aGVHD 1‐2 and no‐aGVHD in terms of MDSCs levels at the time of engraftment, we further analyzed our data according to aGVHD severity (aGVHD 0‐2 vs. aGVHD3‐4, *P *=* *0.0015; aGVHD 0‐2 vs. normal, *P *=* *0.0002). *P* value: *P <* *0.05; **P <* *0.005; ***P <* *0.0001.

Table S1. List of antibodies.
Table S2. The graft content of donors.
Table S3. The mean levels of MDSCs proportion after allo‐HSCT. (a) The mean levels of MDSCs proportion when engraftment. (b) The mean levels of MDSCs proportion after allo‐HSCT.
Table S4. Summary of clinical outcomes.
Table S5. The mean levels of the cytokines in patients. (a) The mean levels of the cytokines grouped by GVHD. (b) The mean levels of the cytokines grouped by MDSC levels.
Table S6. Variables from the Multivariate Analysis Describing the Probability of developing aGVHD.
