# Supplementary figures and images for: Circulating CD14+ HLA‐DR ‐/low myeloid‐derived suppressor cells in leukemia patients with allogeneic hematopoietic stem cell transplantation: novel clinical potential strategies for the prevention and cellular therapy of graft‐versus‐host disease
Source: Cancer Med. 2016 Apr 25;5(7):1654–69. doi: 10.1002/cam4.688 (PMC4944894; doi:10.1002/cam4.688)

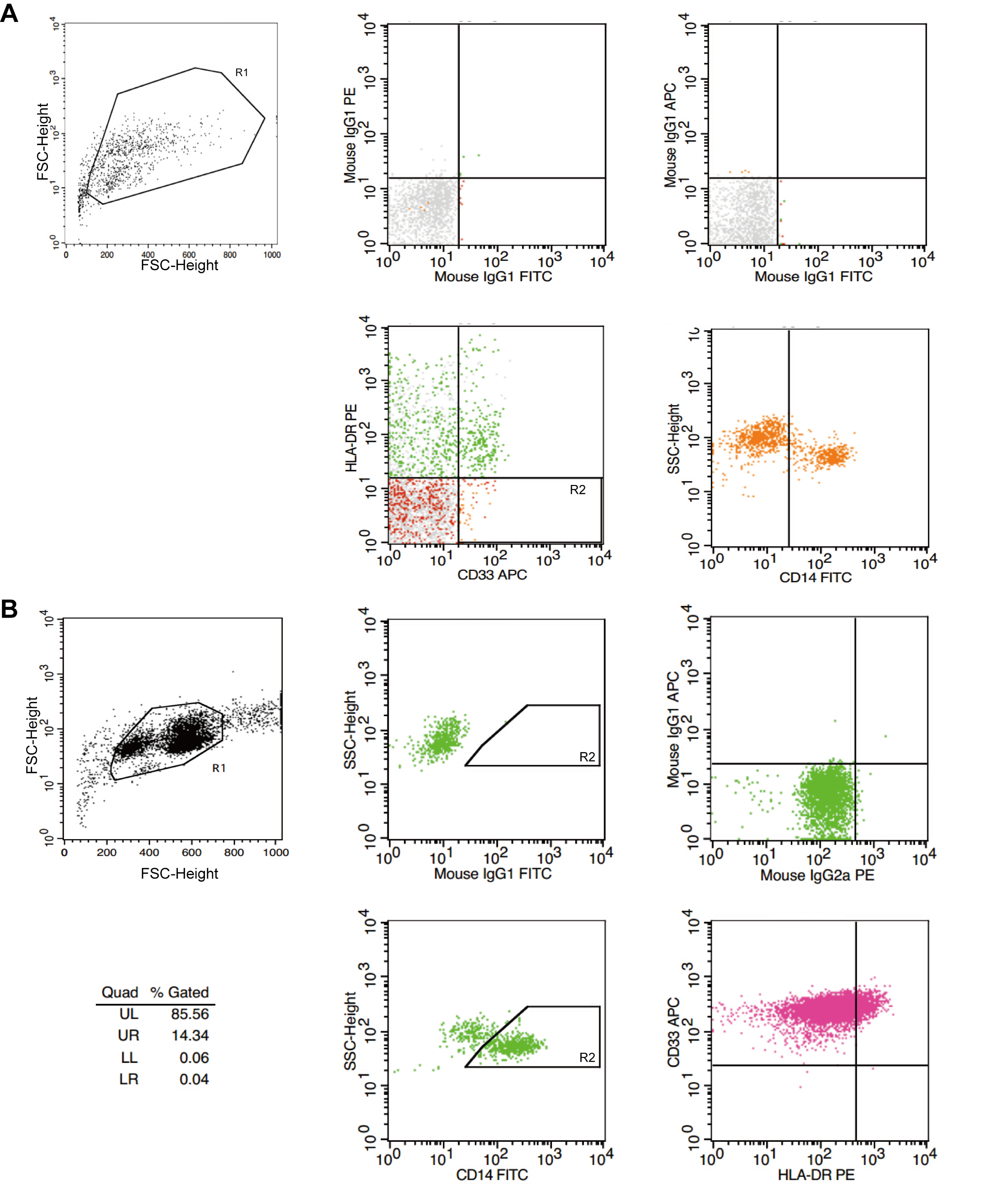

Supplement: Supplementary file 1 — Figure S1. Gating strategy for MDSCs analysis. (A) Flow cytometric analysis of MDSCs in PBMCs of patients. (B) Flow cytometric analysis of MDSCs after purification. [file CAM4-5-1654-s001.tif]

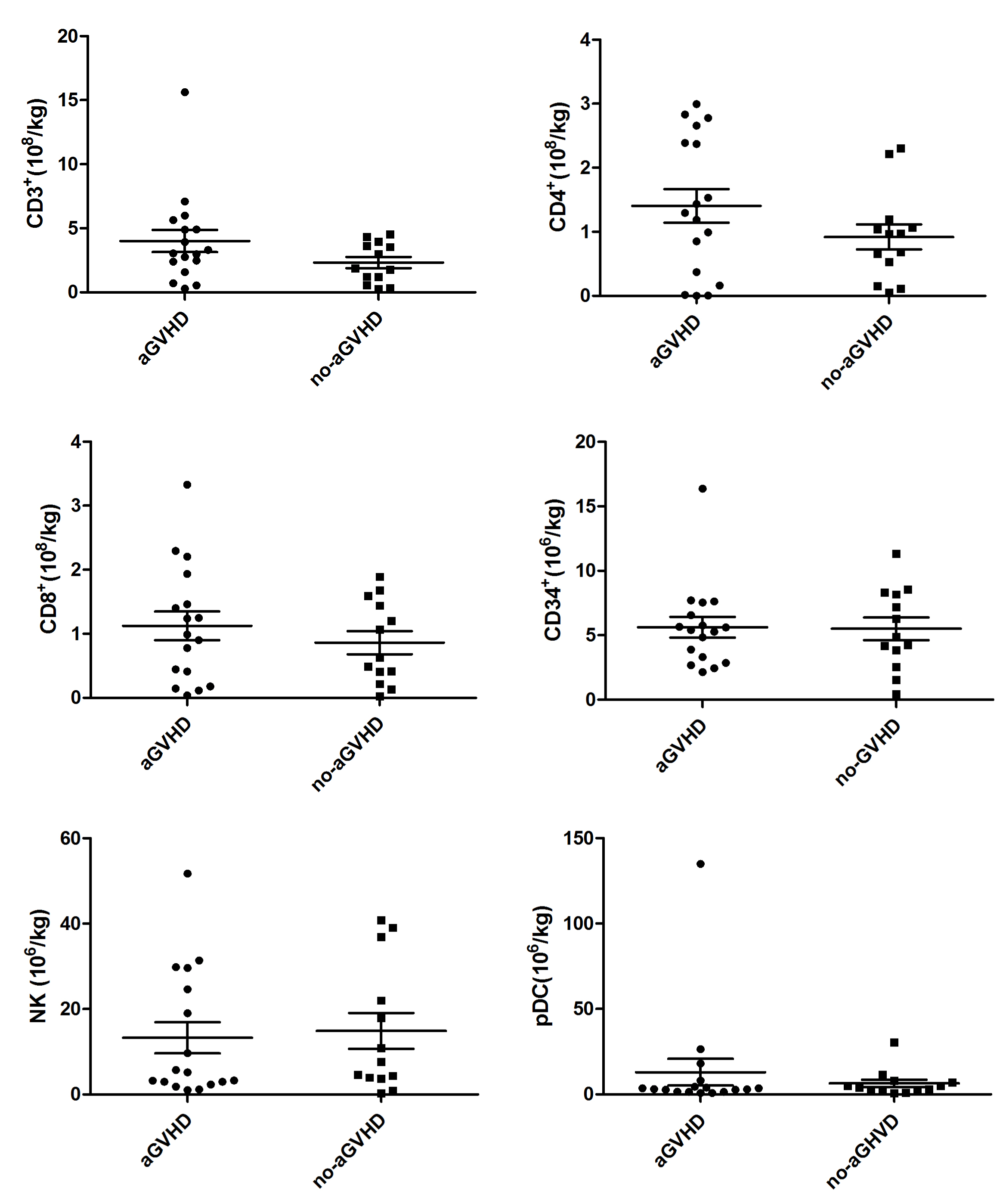

Supplement: Supplementary file 2 — Figure S2. The graft content of different cell population shown to impact on aGVHD was analyzed. No significant correlations were found between the numbers of CD34+ cells, CD3+ T cells, CD3+CD4+T helper cells, CD3+CD8+ cytotoxic T cells, CD3−CD56+ NK cells, and pDC infused and aGVHD development. [file CAM4-5-1654-s002.tif]

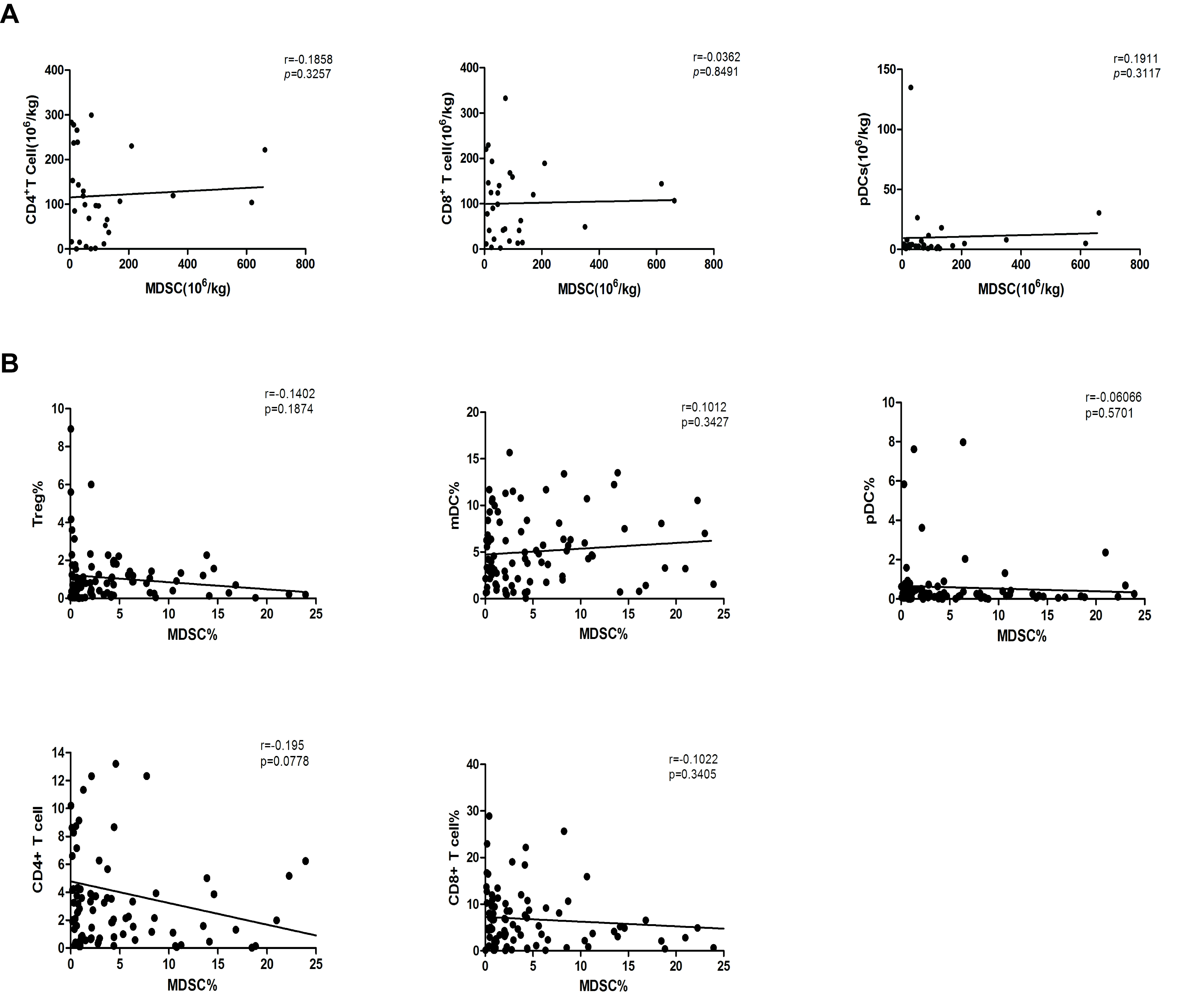

Supplement: Supplementary file 3 — Figure S3. Correlation between the frequencies of MDSCs and other PBMCs subsets. (A) Correlations between the frequencies of MDSCs and other PBMCs subsets in the graft. (B) Correlations between the frequencies of MDSCs and other PBMCs subsets after allo‐HSCT. [file CAM4-5-1654-s003.tif]

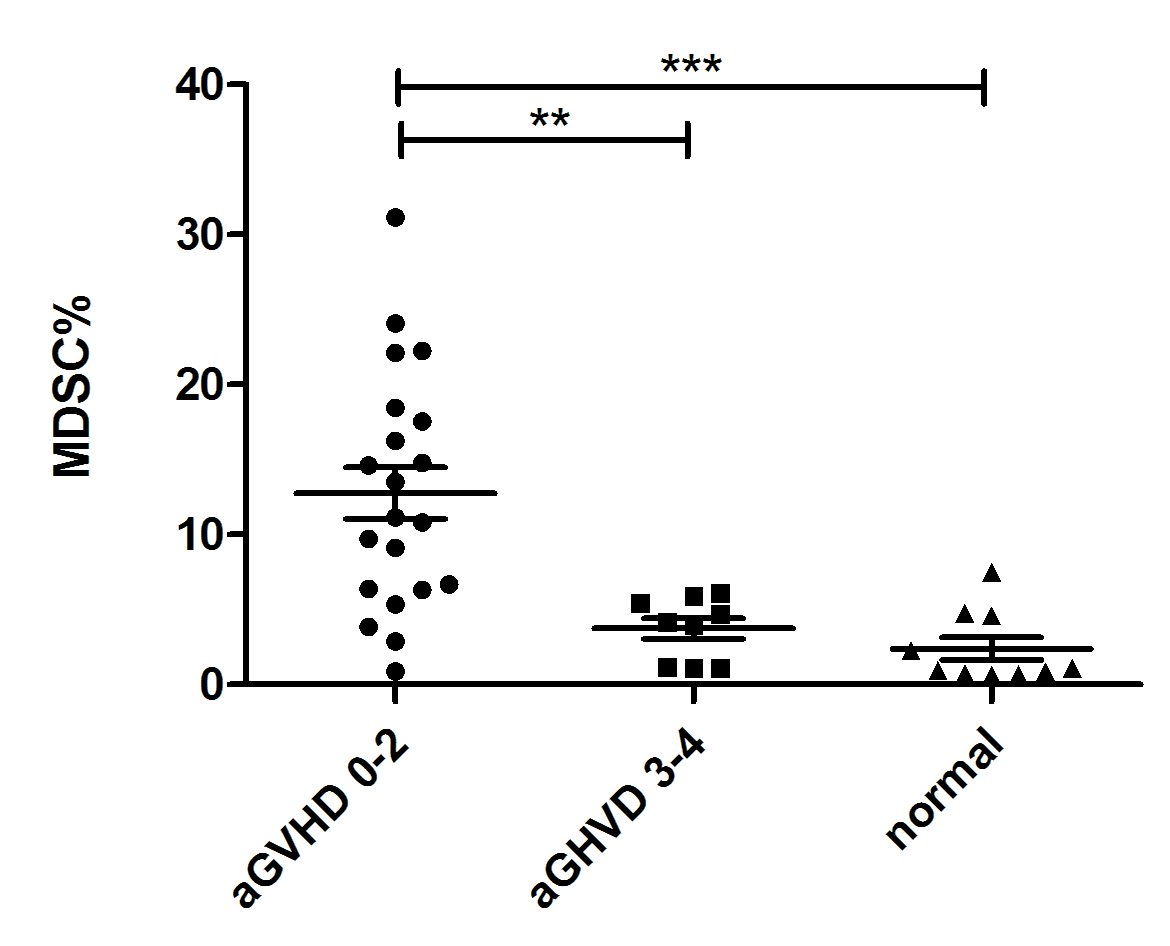

Supplement: Supplementary file 4 — Figure S4. MDSCs frequencies at the time of engraftment. Since no difference was observed between patients with aGVHD 1‐2 and no‐aGVHD in terms of MDSCs levels at the time of engraftment, we further analyzed our data according to aGVHD severity (aGVHD 0‐2 vs. aGVHD3‐4, P = 0.0015; aGVHD 0‐2 vs. normal, P = 0.0002). P value: *P < 0.05; **P < 0.005; ***P < 0.0001. [file CAM4-5-1654-s004.tif]
