# Supplementary material for: Circulating CD14+ HLA‐DR ‐/low myeloid‐derived suppressor cells in leukemia patients with allogeneic hematopoietic stem cell transplantation: novel clinical potential strategies for the prevention and cellular therapy of graft‐versus‐host disease
Source: Cancer Med. 2016 Apr 25;5(7):1654–69. doi: 10.1002/cam4.688 (PMC4944894; doi:10.1002/cam4.688)
Supplement: Supplementary file 5 — Table S1. List of antibodies. Table S2. The graft content of donors. Table S3. The mean levels of MDSCs proportion after allo‐HSCT. (a) The mean levels of MDSCs proportion when engraftment. (b) The mean levels of MDSCs proportion after allo‐HSCT. Table S4. Summary of clinical outcomes. Table S5. The mean levels of the cytokines in patients. (a) The mean levels of the cytokines grouped by GVHD. (b) The mean levels of the cytokines grouped by MDSC levels. Table S6. Variables from the Multivariate Analysis Describing the Probability of developing aGVHD. [file CAM4-5-1654-s005.docx]

**Supplemental Table 1: List of antibodies**

| **Antigen** | **Fluorochrome** | **Clone** |
| --- | --- | --- |
| CD4^1^ | FITC | RPA-T4 |
| CD14^1^ | FITC | M5E2 |
| CD3^1^ | PE | HIT3a |
| FoxP3^2^ | PE | 259D |
| CD11c^2^ | PE | 3.9 |
| HLA-DR^1^ | PE | G46-6 |
| CD8^1^ | APC | RPA-T8 |
| CD25^1^ | APC | M-A251 |
| CD33^1^ | APC | WM53 |
| CD123^1^ | APC | 7G3 |
| HLA-DR^2^ | PerCP | L243 |
| Lineage cocktail^2^ (CD3, CD14, CD19, CD20, CD56) | FTIC | UCHT1,HCD14, HIB19, 2H7, HCD56 |

^1^BD Biosciences, ^2^Biolegend

**Supplemental Table 2. The graft content of donors**

| **Mean±SEM (10^6^/kg)** | aGVHD 1-2 | aGVHD 3-4 | no-aGVHD |
| --- | --- | --- | --- |
| MDSCs (10^6^ /kg) | 61.96±13.67 | 20.37±4.304 | 209.0±57.68 |
| ***p* value** |  | | |
| aGVHD 1-2 |  | *p*=0.0081* | *p*=0.0649 |
| aGVHD 3-4 | *p*=0.0081* |  | *P=*0.0139* |
| no-aGVHD | *p*=0.0649 | *P=*0.0139* |  |

| **Mean±SEM (10^6^/kg)** | aGVHD 1-2 | aGVHD 3-4 | no-aGVHD |
| --- | --- | --- | --- |
| Tregs (10^6^ /kg) | 5.039±2.504 | 1.20±0.4720 | 11.73±2.644 |
| ***p* value** |  | | |
| aGVHD 1-2 |  | *p*=0.1312 | *p*=0.1035 |
| aGVHD 3-4 | *p*=0.1312 |  | *P=*0.0039** |
| no-aGVHD | *p*=0.1035 | *P=*0.0039** |  |

| **Mean±SEM (10^6^/kg)** | aGVHD 1-2 | aGVHD 3-4 | no-aGVHD |
| --- | --- | --- | --- |
| mDCs (10^6^ /kg) | 278.8±56.01 | 367.6±72.94 | 117.0±34.81 |
| ***p* value** |  | | |
| aGVHD 1-2 |  | *p*=0.3589 | *p*=0.0176* |
| aGVHD 3-4 | *p*=0.3589 |  | *P=*0.0027** |
| no-aGVHD | *p*=0.0176* | *p=*0.0027** |  |

*p* value: **p*<0.05; ***p*<0.005; ****p*<0.0001.

**Supplemental Table 3. The mean levels of MDSCs proportion after allo-HSCT**

**a. The mean levels of MDSCs proportion when engraftment**

| **the mean level ±SEM** | aGVHD | no-aGVHD | normal |
| --- | --- | --- | --- |
| MDSCs (%) | 6.148±1.165 | 15.16±2.294 | 2.392±0.756 |
| ***p* value** |  | | |
| aGVHD |  | *p=*0.0017 | *p*=0.0112 |
| no-aGVHD | *p=*0.0017 |  | *p*=0.0002 |
| normal | *p*=0.0112 | *p*=0.0002 |  |

| **the mean level ±SEM** | aGVHD 1-2 | aGVHD 3-4 | no-aGVHD | normal |
| --- | --- | --- | --- | --- |
| MDSCs (%) | 8.856±1.999 | 3.740±0.693 | 15.16±2.294 | 2.392±0.756 |
| ***p* value** |  | | | |
| aGVHD 1-2 |  | *p*=0.0228 | *p*=0.1032 | *p*=0.0085 |
| aGVHD 3-4 | *p*=0.0228 |  | *P=*0.0005 | *p*=0.0789 |
| no-aGVHD | *p*=0.1032 | *P=*0.0005 |  | *p*=0.0002 |
| normal | *p*=0.0085 | *p*=0.0789 | *p*=0.0002 |  |

**b. The mean levels of MDSCs proportion after allo-HSCT**

| **the mean level±SEM** | aGVHD | no-aGVHD | normal |
| --- | --- | --- | --- |
| MDSCs (%) | 7.725±1.460 | 3.423±1.044 | 2.392±0.756 |
| ***p* value** |  | | |
| aGVHD |  | *p*=0.0213 | *p*=0.0084 |
| no-aGVHD | *p*=0.0213 |  | *p*=0.7802 |
| normal | *p*=0.0084 | *p*=0.7802 |  |

| **the mean level ±SEM** | aGVHD 0-2 | aGVHD 3-4 | normal |
| --- | --- | --- | --- |
| MDSCs (%) | 4.269±0.916 | 9.575±2.224 | 2.392±0.756 |
| ***p* value** |  | | |
| aGVHD 0-2 |  | *p*=0.0210 | *p*=0.6270 |
| aGVHD 3-4 | *p*=0.0210 |  | *p*=0.0021 |
| normal | *p*=0.6270 | *p*=0.0021 |  |

**Supplemental Table 4. Summary of clinical outcomes**

| **Clinical outcomes (%)** | **overall** | **graft** | | **PBMCs after allo-HSCT** | |
| --- | --- | --- | --- | --- | --- |
|  |  | **high MDSCs group** | **low MDSCs group** | **high MDSCs group** | **low MDSCs group** |
| 2-year OS | 76.667 | 100 | 50 | 70 | 90 |
| 100d Relapse | 7.200 | 0 | 17.460 | 10.938 | 0 |
| 2-year Relapse | 15.109 | 6.250 | 29.252 | 17.299 | 11.111 |
| 2-year NRM | 21.468 | 0 | 49.519 | 27.449 | 10 |

**Supplemental Table 5. The mean levels of the cytokines in patients**

**a. The mean levels of the cytokines grouped by GVHD**

| **the mean level±SEM** | **aGVHD** | **no-aGVHD** | **normal control** |
| --- | --- | --- | --- |
| **IL-6 (pg/ml)** | 5.059±1.190 | 1.531±0.482 | 0.995±0.290 |
| **IL-10 (pg/ml)** | 23.14±2.719 | 12.60±1.875 | 7.121±1.686 |
| **TNF-a (pg/ml)** | 8.795±1.479 | 3.441±0.853 | 4.633±0.599 |
| **IL-1β (pg/ml)** | 0.574±0.228 | 1.595±1.096 | 0.3056±0.051 |
| **Arg (ng/ml)** | 42.16±4.776 | 18.92±2.942 | 25.93±3.499 |
| **iNOS (ng/ml)** | 31.30±7.257 | 9.499±1.742 | 8.331±1.634 |
| **HO-1 (ng/ml)** | 3.11±0.463 | 1.435±0.327 | 1.225±0.219 |

| ***p* value** | **aGVHD vs. no-aGVHD** | **aGVHD vs. normal** | **no-aGVHD vs. normal** |
| --- | --- | --- | --- |
| **IL-6** | 0.0213* | 0.0053* | 0.1629 |
| **IL-10** | 0.0036* | 0.0001* | 0.0377* |
| **TNF-a** | 0.0017* | 0.0347* | 0.0865 |
| **IL-1β** | 0.1481 | 0.9799 | 0.0506 |
| **Arg** | 0.0024* | 0.0112* | 0.1629 |
| **iNOS** | 0.0026* | 0.0046* | 0.7097 |
| **HO-1** | 0.0050* | 0.0129* | 0.8040 |

**b. The mean levels of the cytokines grouped by MDSC levels**

| **the mean level±SEM** | **high MDSCs group** | **low MDSCs group** | **normal control** |
| --- | --- | --- | --- |
| **IL-6 (pg/ml)** | 4.909±1.181 | 1.955±0.788 | 0.995±0.290 |
| **IL-10 (pg/ml)** | 17.76±1.809 | 19.50±3.757 | 7.121±1.686 |
| **TNF-a (pg/ml)** | 8.991±1.609 | 4.744±1.181 | 4.633±0.599 |
| **IL-1β (pg/ml)** | 1.599±0.898 | 0.3508±0.148 | 0.3056±0.0514 |
| **Arg (ng/ml)** | 41.07±5.387 | 21.91±3.130 | 25.93±3.499 |
| **iNOS (ng/ml)** | 31.48±7.674 | 10.85±2.332 | 8.331±1.634 |
| **HO-1 (ng/ml)** | 3.130±0.500 | 1.531±0.301 | 1.225±0.219 |

| ***p* value** | **high MDSCs group vs.**  **low MDSCs group** | **high MDSCs group vs. normal control** | **low MDSCs group vs. normal control** |
| --- | --- | --- | --- |
| **IL-6** | 0.0134* | 0.0017* | 0.2787 |
| **IL-10** | 0.7083 | 0.0011* | 0.0049* |
| **TNF-a** | 0.0142* | 0.0417* | 0.3948 |
| **IL-1β** | 0.4169 | 0.8121 | 0.1204 |
| **Arg** | 0.0178* | 0.0373* | 0.5007 |
| **iNOS** | 0.0106* | 0.0084* | 0.4641 |
| **HO-1** | 0.0188* | 0.0114* | 0.7474 |

High MDSCs group:MDSCs proportion >2.798% ;

Low MDSCs group: MDSCs proportion≤2.798%.

**Supplemental Table 6. Variables from the Multivariate Analysis Describing the Probability of developing aGVHD.**

| **Variables** | ***p*** |
| --- | --- |
| sex | 0.367 |
| age | 0.16 |
| CD3^+^ cells | 0.117 |
| CD4^+^ cells | 0.163 |
| CD8^+^ cells | 0.37 |
| CD34^+^ cells | 0.914 |
| NK cells | 0.765 |
| MDSCs | 0.004** |
